# Drug response in the era of precision medicine: A methodological review

**DOI:** 10.1016/j.csbj.2025.11.067

**Published:** 2025-12-02

**Authors:** Daniella Okyere, Laura Bravo-Merodio, Yuanwei Xu, Xin Guan, Durga Parkhi, Georgios Gkoutos, Animesh Acharjee

**Affiliations:** a Cancer and Genomic Sciences, University of Birmingham, Birmingham, UK; b Centre for Health Data Research, University of Birmingham, Birmingham, UK; c Institute of Translational Medicine, University Hospitals Birmingham NHS, Foundation Trust, B15 2TT, UK

**Keywords:** Drug response, machine learning, multomics

## Abstract

The growing availability of structured data types, including molecular and pharmacological data, along with unstructured data types such as medical imaging data, has enabled the development of statistical, machine learning (ML), and deep learning (DL) approaches for drug response prediction. These computational methods are integral to precision medicine, leveraging data-driven techniques to predict patient-specific treatment outcomes. This review provides a systematic overview of existing methodologies for drug response prediction, focusing on input data structures, response variable definitions, and data types utilized. In contrast to previous reviews that focus on specific therapies or computational approaches, we present a unified classification framework based on data-response relationships, including single data type with a response vector, single data type with a response matrix, and multiple data types with a response. By using this structure, we can compare statistical and ML-based models across different diseases and data types. Finally, we discuss evaluation strategies, highlight emerging methodological trends, and outline key challenges and future opportunities to advance drug response prediction.

## Graphical Abstract

**Figure:**
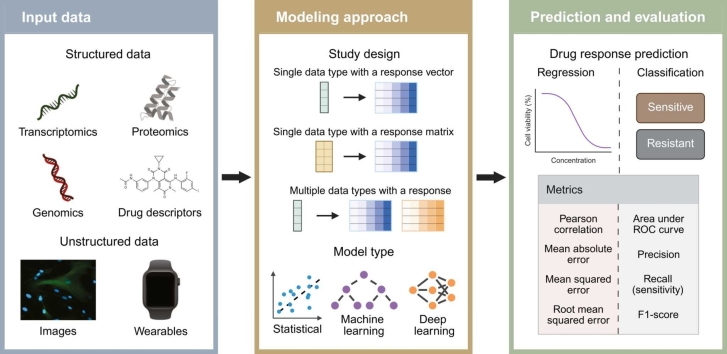

## 1 Introduction

The term "drug response" pertains to the physiological and biochemical reactions exhibited by an individual's body in response to a certain medicine, drug, combination, or therapeutic intervention [1]. The response to medications can vary among individuals as responders or non-responders due to multiple factors. Those factors can broadly be classified into two aspects: molecular and phenotypic traits. Molecular traits can include genetic variants, gene expression, epigenetics, protein or metabolite levels, and microbiome diversity, etc. The other phenotypic labels can include environmental circumstances, demographics, lifestyle, ethnicity, age, gender, diet, other medications, etc.

The comprehension of medication response between populations as responders or non-responders is vital in order to enhance treatment efficacy, cost-effective treatment [2], optimize therapeutic results [3], [4], and mitigate the occurrence of adverse events [5]. Achieving this goal is challenging because patients exhibit significant variability driven by genetic differences, disease heterogeneity, and molecular as well as environmental factors [6]. Precision medicine addresses these complexities by tailoring treatments to each patient’s genetic, molecular, and clinical profile [7], a strategy that has already transformed therapeutic approaches across many diseases. By identifying key molecular differences among patients, researchers have developed targeted treatments that outperform conventional one-size-fits-all approaches. Notable examples include HER2-targeted therapies in breast cancer [8], BRAF inhibitors for melanoma [9], and KRAS-directed treatments for colorectal cancer [10]. These developments show how the characterization of specific genetic alterations, including gene variants and fusions, across diverse cancer types has broadened the scope and impact of personalized medicine.

To build on these advances, recent years have witnessed the generation of vast biomedical datasets through efforts ranging from individual research laboratories to multinational collaborative consortia. These resources form the backbone of precision medicine by enabling large-scale analyses that integrate genomic, transcriptomic, proteomic, and pharmacogenomic data. Among the most widely used for drug response prediction—particularly in cancer research—are the Genomics of Drug Sensitivity in Cancer (GDSC) [11], [12], Connectivity Map [13], and Cancer Cell Line Encyclopedia (CCLE) [14]. Molecular representations such as Morgan fingerprints and SMILES further support computational modeling and predictive analysis. More recent platforms, including the Cancer Dependency Map [15], NCI Cancer Research Data Commons, and Human Tumor Analysis Network, provide harmonized, multi-omics datasets and physiologically relevant models [16]. Interactive tools like cBioPortal facilitate exploration of curated cancer genomics, while population-level resources such as PopTradeOff highlight the importance of genetic diversity in predictive modeling [17]. Beyond traditional cell lines, patient-derived xenografts (PDXs), organoids, and large-scale clinical datasets (e.g., TCGA) offer clinically relevant models for drug testing. Collectively, these resources expand the scope of precision medicine by supporting comprehensive and translational studies of drug response, driving advances in patient stratification, diagnostic accuracy, and therapeutic innovation [18].

Given the omics data acquired from cell lines or tumors, along with their phenotypic drug responses, various computational frameworks have emerged to predict treatment outcomes. Drug response prediction is typically framed as either a classification task, categorizing responses into discrete groups like responsive or non-responsive, or a regression task, estimating continuous outcomes such as IC50 or other pharmacological metrics. These frameworks encompass statistical approaches and artificial intelligence (AI)-driven models—such as machine learning (ML) and deep learning (DL)—applied across diverse therapeutic domains. Statistical methods, such as logistic regression and survival analysis, have traditionally been used to model relationships between clinical outcomes and molecular predictors [19]. Traditional ML techniques, including Random Forest (RF) [20], Support Vector Machines (SVM) [21], and ensemble methods, extend these capabilities by learning complex, non-linear patterns from high-dimensional data. More recently, DL approaches, such as neural networks, autoencoders [22], [23], convolutional neural networks (CNNs) [24], [25], and graph neural networks (GNNs) [26], [27], [28], [29], have demonstrated superior performance in capturing intricate biological relationships and integrating heterogeneous data types. Collectively, these methodologies aim to improve predictive accuracy, enable robust patient stratification, and guide precision medicine strategies (Table 1 summarizes key methods referenced throughout this review).

**Table 1:** Overview and brief descriptions of computational methods discussed in this review. This table summarizes key statistical, machine learning, and deep learning approaches covered in the review, offering concise descriptions of each method along with relevant references for further reading.

**Category**	**Method**	**Description**	**More information**
Statistical methods	Analysis of covariance	Assesses the differences in group means while controlling for the effects of a continuous covariate that may influence the outcome.	[136]
	Logistic regression	Technique for predicting binary outcomes (e.g., 0 = absence, 1 = presence) by modeling the probability of the event using a Bernoulli (binomial) distribution and estimating parameters via maximum likelihood for classification.	[137]
	Paired *t*-test	Compares the means of two related (paired) groups to determine whether their population means differ. It assumes that the differences between paired observations are normally distributed.	[138], [139]
	Survival analysis	A collection of statistical methods for analyzing the time until an event occurs (e.g., death, relapse, recovery). These techniques account for censoring, where the event time is not fully observed, and typically assume that censoring is uninformative.	[140]
	Wilcoxon signed-rank test	A nonparametric method used to compare two independent samples to determine whether they originate from populations with the same median. This statistical test does not assume normality but requires that the distributions have similar shapes.	[141]
Traditional machine learning methods	Principal component analysis (PCA)	Multivariate statistical technique that reduces the complexity of high-dimensional data into a smaller number of uncorrelated components, capturing the maximum variance in the data.	[142]
	Random Forest (RF)	Method that builds multiple decision trees through bootstrap aggregation and random predictor selection to improve model accuracy and reduce overfitting.	[20]
	Support Vector Machine (SVM)	Algorithm that identifies the optimal hyperplane with the largest margin to separate observations of different classes within a transformed feature space.	[143], [144]
Deep learning methods	Artificial neural network	A collection of interconnected nodes (artificial neurons) organized into layers inspired by the structure of the human brain. These layers process input data and learn complex patterns through iterative weight adjustments, enabling tasks such as classification, prediction, and pattern recognition.	[145], [146]
	Autoencoder	A type of neural network composed of an encoder and a decoder that learns compact, lower-dimensional representations of input data. Using unsupervised learning and backpropagation, autoencoders automatically capture salient features without requiring labeled data or manual feature engineering.	[147]
	Convolutional neural network (CNN)	Multi-layered neural networks that automatically learn and extract features from data for tasks such as image and speech recognition, natural language processing, and classification.	[148], [149]
	Graph neural network (GNN)	Neural network architectures within deep learning that capture relationships between nodes in a graph through message passing mechanisms.	[150], [151]
	Variational autoencoder (VAE)	Method based on a probabilistic framework used to learn nonlinear latent representations that generalize to unseen data.	[152]

Artificial intelligence is transforming precision medicine [30] by enabling more individualized treatment strategies across oncology and chronic disease management. In cancer therapy, frameworks such as NeurixAI leverage transcriptomics-informed DL to model drug–gene interactions, predict treatment response, uncover novel anticancer properties, and identify resistance drivers through explainable AI (NeurixAI Consortium, 2024). These findings highlight the growing power of integrating multi-omics with xAI for personalized therapy selection and drug repurposing. Beyond cancer, AI enhances chronic disease care—for example, ML and DL models trained on large-scale clinical data reliably forecast drug efficacy in type 2 diabetes, with ensemble models showing particularly strong predictive accuracy [31]. AI also addresses persistent challenges such as drug resistance by characterizing mechanisms, predicting resistant phenotypes, and informing adaptive combination therapies [32]. Furthermore, AI accelerates early drug discovery through drug–target interaction prediction, reducing experimental burden and prioritizing candidates based on binding affinity estimates. While these advances promise transformative impact, maintaining methodological rigor is essential to ensure results are robust, unbiased, and reproducible—principles emphasized by the NIH as critical for translating evidence into individualized patient care [33].

Previous reviews have examined drug response prediction, but most focused on specific methodological or biological contexts. For instance, one review emphasized monotherapy prediction in personalized oncology [34], while others explored molecular representation learning [35], DL-based prediction frameworks [4], single-cell drug activity inference [36], combination drug response modeling [37], [38], and immune checkpoint inhibitor response prediction [39]. Broader reviews have addressed computational modeling in precision medicine, highlighting multi-omics integration, multimodal data analysis, and advanced ML/DL approaches for disease characterization and patient stratification [40], [41], [42], [43]. By situating our review within both drug response–specific contexts and the wider computational landscape, we aim to provide an integrative perspective that spans diverse diseases and data modalities, outlining unifying trends, methodological innovations, and key directions for future research.

**Fig. 1:**
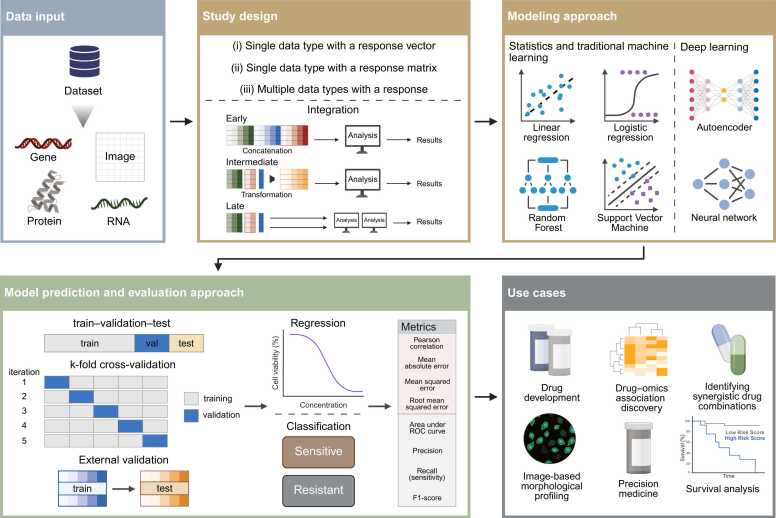
Integrated overview of data modalities, study designs, modeling strategies, evaluation frameworks, and application domains in drug response prediction. The data input panel depicts the range of structured data (e.g., transcriptomics, genomics, proteomics) and unstructured data (e.g., imaging) commonly used in drug response studies. The study design panel summarizes study designs—single data type with a response vector, single data type with a response matrix, and multi-modal designs—together with integration strategies for multi-omics analyses. The modeling approach panel highlights representative computational paradigms spanning statistical methods, traditional machine learning algorithms, and deep learning architectures. The evaluation framework panel outlines major validation strategies, including train–validation–test splits, k-fold cross-validation, and external validation, and organizes performance metrics by task type, with regression metrics shown in red and classification metrics in green. The use cases panel illustrates translational applications such as precision medicine and drug–omics association analysis.

In this review, we examine the use of structured and unstructured data in drug response prediction, focusing on how different methods and data types influence predictive performance. We classify studies into three categories: (i) single data type with a response vector, (ii) single data type with a response matrix, and (iii) multiple data types with a response. A response vector links one data matrix (e.g., gene expression) to a single outcome per sample, whereas a response matrix associates one data matrix with multiple outcomes, enabling multi-task learning. In contrast, multi-modal approaches integrate heterogeneous data matrices with either a response vector or matrix (Fig. 1). This classification highlights the strengths and limitations of each approach. Finally, we discuss key challenges, emerging methodological trends, and future research directions aimed at improving predictive accuracy and enhancing model generalizability in clinical settings.

## 2 Methods

Our review employed a structured, multi-stage approach to identify studies applying statistical, ML, or DL methods for drug response prediction across both monotherapy and combination therapy contexts:

1. Literature search: we queried PubMed, Scopus, and Google Scholar using combinations of keywords, including drug response prediction, drug sensitivity prediction, monotherapy, drug combinations, synergy prediction, multi-omics, statistical methods, machine learning, and deep learning. No restrictions were placed on publication year; however, priority was given to recent studies to capture current methodological advances.
2. Screening and eligibility: titles and abstracts were screened to identify studies employing computational modeling for predicting drug response, drug synergy, or drug-associated phenotypes. Studies limited to experimental pharmacology without predictive modeling were excluded. Full-text articles passing the initial screen were assessed for eligibility.
3. Data extraction and structured assessment: each eligible study was reviewed using a standardized framework. Specifically, we recorded whether the study utilized structured data and/or unstructured data. We also categorized the study design as one of the following: single data type with a vector response, single data type with a matrix response, or multiple data types with a response. Finally, we noted the modeling approach employed, distinguishing between statistical methods, traditional ML algorithms, and DL architectures. Additional details extracted included datasets used, prediction tasks (regression, classification, synergy scoring), methodological frameworks, integration strategies, evaluation procedures (e.g., train–validation–test split, cross-validation, external validation), and code availability.
4. Synthesis and categorization: included studies were synthesized and classified according to data format and study design.

### 2.1 Structured data

In this section, we consider structured data, which refers to information that is organized according to a predefined schema or model, typically stored in relational databases or spreadsheets. Within this framework, data elements are arranged in fixed fields with defined types and relationships, which enhances their searchability, interoperability, and suitability for computational analysis. Structured data can include both quantitative and qualitative variables and is widely used in biomedical research. Table 2 summarizes studies using structured data types and their corresponding designs, with additional details provided in Supplementary Table 1.

**Table 2:** Summary of methods for drug response prediction and phenotypic profiling across biomedical datasets.

**Paper**	**Dataset**	**Task**	**Split strategy**	**Evaluation metric**	**External test protocol**	**Fixed random seeds**	**Code availability**
**Single data type with a response vector**							
Le et al. (2017)	Clinical trial dataset (Epidemiological data)	Clinical drug-response assessment using RECIST/immune-related criteria; survival analysis via Kaplan–Meier and log-rank test	-	Radiographic objective response rate, complete response rate, progression‑free survival, overall survival	-	-	-
Costello et al. (2014)	NCI-DREAM challenge (multi-omics + drug response profiles)	Drug sensitivity prediction using kernel methods, nonlinear regression (regression trees), sparse linear regression, PLS/PC regression, ensemble approaches	Held-out test cell lines: 35 for training, 18 for testing	Weighted probabilistic c-index (primary), probabilistic c-index, scaled weighted probabilistic c-index, SRCC	Cross-dataset (GDSC)	Yes	https://www.nature.com/articles/nbt.2877#MOESM3
Chen et al. (2022)	GDSC, CCLE (Bulk RNA-seq), GEO (scRNA-seq)	Single-cell drug response prediction using transfer learning from bulk RNA-seq	Train–validation–test on bulk RNA-seq; scRNA-seq used for cross-domain evaluation	F1-score, AUROC, AP score, precision, recall, AMI, Adjusted Rand Index	-	Yes	https://github.com/OSU-BMBL/scDEAL
Zhang et al. (2018)	GDSC (gene expression)	Binary classification of cell-line drug sensitivity using a network-based method	LOOCV	AUROC	-	-	https://github.com/USTC-HIlab/HNMDRP
Tan (2016)	GDSC, CCLE, NCI-DREAM (gene expression)	Drug sensitivity prediction using multi-task learning	Random 90 %/10 % train–test split	MSE (predicted vs observed IC50 values)	-	-	http://mtan.etu.edu.tr/Supplementary/kMTrace/
Beck et al. (2017)	Clinical trial dataset (digital health)	Assessment of effectiveness of real-time continuous glucose monitoring versus usual care on glycemic control	Randomised 1:1 allocation to continuous glucose monitoring vs usual care groups	Adjusted difference in mean glycated hemoglobin change	-	-	-
Loo, Wu & Altschuler (2007)	High-content imaging (100 compounds; ∼300 phenotypic features/cell)	Cancer cell classification using support vector machine method; drug effect size and phenotypic shift quantification	-	Classification score of drug effect, phenotypic shift (vector)	-	-	-
Lu et al. (2019)	High-content imaging (∼3000,000 single-cell crops from yeast ORF-GFP collection; 4049 proteins, ∼778 crops per protein)	Paired cell inpainting with a self-supervised CNN to learn features for downstream tasks (protein localisation, clustering, functional group discovery)	LOOCV	Classification accuracy	-	-	https://github.com/alexxijielu/paired_cell_inpainting
Ando, McLean & Berndl (2017)	BBBC021 (high-content imaging)	phenotypic discrimination using a Deep Metric Network	-	Accuracy (not same compound/not same compound or batch)	-	-	-
Kraus, Ba & Frey (2016)	High-content imaging (one mammalian high-content screen and one yeast protein-localization screen)	Simultaneous image-level classification (phenotype/treatment class) and segmentation using deep CNNs with multiple-instance learning	Train–test split (dataset-specific)	Accuracy	-	-	-
Fang et al. (2024)	GEO (gene expression)	Prognosis prediction in cervical cancer using a hypoxia-related gene signature (Cox and LASSO regression), survival analysis (Kaplan-Meier)	KFCV	AUROC, Hazard ratios (survival risk stratification)	-	-	-
Wang et al. (2023)	Somatic mutation and clinical outcome data from previous studies and TCGA melanoma	Classify lower-grade glioma into stemness subtypes and predict drug sensitivity	External-cohort validation	Overall survival, AUROC	Cross-dataset (two external cohorts)	-	-
Zhou et al. (2023)	TCGA, GEO (Bulk RNA-seq and scRNA-seq)	Classify lower-grade glioma into stemness-based subtypes using mRNAsi/GSScore and ML; predict prognosis via Kaplan-Meier analysis	train–test	Overall survival, AUROC	Cross-dataset (CGGA)	-	-
Hu et al. (2024)	GEO (Bulk RNA-seq, scRNA-seq)	Classification and characterizaion of PANoptosis patterns in psoriasis via unsupervised clustering	-	-	-	-	-
Zheng et al. (2023)	GDSC (bulk RNA-seq), Broad Institute's single-cell portal (scRNA-seq)	Single-cell drug response prediction via domain transfer learning	KFCV, scRNA-seq as target for cross-domain evaluation	Average AUROC and AUPR	-	Yes (evaluated with multiple random seeds for robustness)	https://github.com/CompBioT/SCAD
Liu, Duan & Luo (2024)	GDSC (bulk RNA-seq), GEO (scRNA-seq)	Single-cell drug response prediction using multi-source domain transfer learning	Bulk RNA-seq cell lines as source domains, scRNA-seq as target for cross-domain evaluation	AUROC, AUPR	-	-	https://github.com/hliulab/scAdaDrug
**Single data type with a response matrix**							
Kuenzi et al. (2020)	CTRPv2, GDSC (Mutational profiles)	Drug response and synergy prediction using neural networks	Train–validation–test, KFCV	SRCC, AUC, synergy prediction performance	-	-	https://github.com/idekerlab/DrugCell
Jia et al. 2021	CCLE, GDSC (gene expression)	Drug response prediction (ln(IC₅₀)/ActArea regression) using a latent-representation model	KFCV	PCC	Cross-dataset (TCGA and other clinical datasets)	Yes	https://github.com/bsml320/VAEN/
Owens et al. (2021)	Clinical trial dataset (digital health)	Statistical analysis of treatment effects on sleep outcomes in heart failure and reduced ejection fraction patients	Randomised controlled trial (1:1 allocation)	-	-	-	-
Khandwalla et al. (2021)	Clinical trial dataset (digital health)	Assessment of drug impact on activity and sleep via wearable sensors (statistical analysis)	Randomised controlled trial (1:1 allocation)	-	-	-	-
Santo et al. (2019)	Clinical trial dataset (digital health)	Medication-adherence intervention for coronary heart disease using a reminder app	Randomised controlled trial (app intervention vs control)	Medication adherence at 3 months (primary), changes in low-density lipoprotein and blood pressure (secondary)	-	-	-
Liu et al. (2018)	Clinical trial dataset (digital health)	Remote monitoring and management of treatment-related hypertension and diarrhea via mobile app and web portal	-	Usability, acceptability, perceived satisfaction (implementation outcomes)	-	-	-
Orri et al. (2014)	Clinical trial dataset (digital health)	Assessment of treatment efficacy and safety of tolterodine ER in a web‑based clinical trial	Randomized parallel‑group allocation (double-blind)	Change in urinary frequency (primary endpoint), safety/tolerability outcomes	-	-	-
Ben-David et al. (2018)	Images generated using Cell Painting	Assessment of clonal heterogeneity and drug-response variability via unsupervised clustering	-	Clustering concordance with with copy number alteration patterns and genomic divergence	-	-	-
Kümmel et al. (2012)	High-content imaging	Phenotypic clustering of compounds using self-organizing maps	-	Biological relevance of clusters	-	-	-
Dürr and Sick (2016)	BBBC022v1 (high-content imaging)	Single-cell phenotype classification using linear discriminant analysis, Random Forest, support vector machine, and CNN	train–validation–test split	Classification accuracy, log loss	-	-	-
Pawlowski et al. (2016)	BBBC021 (high-content imaging)	Mechanism-of- action prediction using generic deep CNNs	Leave-one-compound-out cross-validation	Classification accuracy	-	Yes	https://github.com/carpenterlab/2016_pawlowski_mlcb
Godinez et al. (2017)	BBBC013, BBBC014–016, BBBC021, WND-CHARM collection, Human Protein Atlas (high-content imaging)	Classify cellular images into phenotypes using a multi-scale CNN	train–test split, leave-one-compound-out cross-validation	Classification accuracy	-	Yes	https://academic.oup.com/bioinformatics/article/33/13/2010/2997285#supplementary-data
Ljosa et al. (2013)	BBBC021 (high-content imaging)	Mechanism-of- action prediction via unsupervised profiling and distance-based classification	Leave-one-compound-out cross-validation	Classification accuracy	-	-	https://pubs.broadinstitute.org/ljosa_jbiomolscreen_2013/
Adams et al. (2006)	Cell imaging	Mechanism-of- action prediction using minimum squared-distance classifier	LOOCV	Classification accuracy	-	-	-
**Multiple data types with a response**							
Sakellaropoulos et al. (2019)	GDSC, TCGA (gene expression)	Drug sensitivity prediction using neural network-based method	KFCV	MSE, MAE, PCC (predicted vs observed IC50)	Cross-dataset (clinical trial + external cohort datasets)	-	https://github.com/TeoSakel/deep-drug-response
Ammad-ud-dinet et al. (2017)	GDSC, FIMM (gene expression, prior knowledge)	Bayesian multi-view/multi-task regression for drug sensitivity prediction	LOOCV	IC50, tailored drug sensitivity score (TDSS)	-	Yes	https://github.com/suleimank/mvlr
Ma et al. (2021)	GDSC, CCLE, DepMap, PDTC BioBank, PDXE (gene expression, somatic mutation)	Few-shot learning-based drug response prediction	Few-shot train–test splits per drug	PCC, SRCC	-	Yes	https://github.com/idekerlab/TCRP/
Chawla et al. (2022)	CCLE, GDSC, CTRPv2 (bulk RNA-seq, scRNA-seq)	Drug sensitivity prediction using deep neural networks	Cell line-based split	PCC (predicted vs observed IC50)	Cross-dataset (TCGA)	Yes	https://github.com/SmritiChawla/Precily
Koukouli et al. (2021)	GDSC (cancer cell line gene expression and dose-response data)	Dosage-dependent drug response regression	KFCV on cell line–drug units, leave-cell-line-out CV	RMSE, MAE for predicted dose–response curve	Cross-dataset (GDSC2) Note: partially overlapping drug sets	Yes	https://github.com/koukoulEv/fbioSelect
Zhao et al. (2023)	Zhu et al. (2022) benchmark dataset (CCLE) — includes gene expression, somatic mutation, and CNV data	Deep learning-based drug response prediction (IC50 regression)	Random split, additional leave-one compound-out and leave-cell-line-out tests with 20 % held-out drugs or cell lines	RMSE, MAE, PCC	Cross dataset (independent dataset from Peng et al., 2022)	-	https://github.com/xyzhang-10/MSDRP
Wang et al. (2021)	CCLE, GDSC (multi-omics: mRNA expression, mutations, CNV, RPPA expression, and metabolite expressions)	Predicting drug response (log IC50) in cancer cell lines (Deep neural network)	KFCV	MSE, R-squared	-	-	-
Chang et al. (2018)	CCLP, GDSC (gene expression, chemical features, and drug-response)	Drug response prediction (ln(IC₅₀) regression) and binary classification (CNN-based models)	train–test, KFCV	R-squared, RMSE, AUROC	-	-	-
Sharma et al. (2023)	GDSC (multi-omics: gene expression, copy-number alteration (CNA) and mutation data converted into image format)	Drug response prediction with CNNs	Training set split further intro train-validation90 %/10 %	AUROC, accuracy, sensitivity, specificity, F1-score	Cross-datasets (PDXE and TCGA)	Yes	https://github.com/alok-ai-lab/DeepInsight3D_pkg
Zhu et al. (2022)	GDSC2, CCLE, COSMIC (gene expression, somatic mutation, CNV)	Drug response prediction using graph neural networks with similarity augmentation	train–validation–test, leave-drug/cell-line-out, KFCV	RMSE, MAE, PCC	-	Yes	https://github.com/violet-sto/TGSA
Park, Lee & Nam (2023)	CCLE, GDSC (gene expression, mutation profiles)	Drug response prediction for individual drugs using deep learning vs classical ML models	train–test split 80 %/20 %	RMSE, R-squared	-	-	https://github.com/labnams/IC50_individual_drug
Allesøe et al. (2023)	In-house generated multi-omics, clinical dataset	Drug–omics association discovery for 20 common type 2 diabetes drugs (Logistic regression)	train–test split	RMSE, MAE, PCC	-	Yes	https://github.com/RasmussenLab/MOVE/
Rohban et al. (2017)	Images generated using Cell Painting assay	Image-based morphological profiling to link gene/allele perturbations with cellular function (morphological signatures) using clustering methods	-	Percentage of genes with detectable morphological profiles, clustering of profiles by biological pathway.	-	Yes	https://github.com/carpenterlab/2017_rohban_elife
Li et al. (2024)	GDSC2 and CellMiner (drug response datasets)	Zero-shot drug-response prediction (domain adaptation for unseen drugs)	Random 80 %/20 % drug split: source (train/validate), target (test)	RMSE, PCC, SRCC, Margin Ranking Loss (Rank)	-	Yes	https://github.com/DrugD/MSDA
Gill et al. (2024)	Clinical trial (Heart rate and physical intervals)	Predict functional class (NYHA class) from wearable sensor data using a self-supervising convolutional neural network	Hold-out patient set, repeated KFCV	F1-score	-	-	https://github.com/gkoutos-group/wearable_data_embedding
Yang, Lin & Sun (2023)	Clinical data of glioma patients (scRNA-seq, spatial transcriptomics, bulk RNA-seq, clinical profiles, and somatic mutation data)	Survival analysis (Kaplan-Meier analysis)	Multiple simulation replicates	Progression-free survival	-	-	-
Shmatko et al. (2025)	UK Biobank (ICD-10 top-level diagnostic codes, Demographic information, lifestyle data)	Modeling the progression and competing risks of over 1000 diseases (attention-based transformer models)	train–validation–test	AUROC	Cross-cohort (Danish registry data)	Yes	https://github.com/gerstung-lab/delphi

Abbreviations: AMI, Adjusted Mutual Information; AUROC, Area under the receiver operating characteristic curve; AUPR, Area under the precision-recall curve; BBBC, Broad Bioimage Benchmark Collection; CCLP, Cosmic Cell Line Project; CNV, copy number variation; c-index, concordance-index; CTRP, Cancer Therapeutics Response Portal; GDSC, Genomics of Drug Sensitivity in Cancer; GEO, Gene Expression Omnibus; KFCV, k-fold cross-validation; LOOCV, Leave-one-out cross-validation; MAE, mean absolute error; MSE, mean squared error; NCI-DREAM, National Cancer Institute Dialogue for Reverse Engineering Assessments and Methods; OR, odds ratio; PCC, Pearson correlation coefficient; PC, principal component; PDXE, PDX Encyclopedia; PLS, partial least squares; pc-index, probabilistic concordance index; RNA-seq, RNA sequencing; RMSE, root mean squared error; R-squared, coefficient of determination; scRNA-seq, single-cell RNA sequencing; SRCC, Spearman’s rank correlation coefficient.

#### 2.1.1 Single data type with a response vector

Cell line experiments have been frequently used for drug response prediction, particularly in studies that analyze a single data type with a vector-based response. Moreover, cell line datasets are predominantly sourced from open data repositories, including GDSC [11], [12], CCLE [14] and TCGA [44], with the greatest number cell line datasets collected from GDSC and CCLE [45]. For example, a recent study integrated bulk RNA sequencing (RNA-seq) datasets from GDSC and CCLE databases, based on shared gene features, to predict single-drug response classifications for cancer and other diseases [46]. This analysis was performed using single-cell Drug rEsponse AnaLysis, a supervised DL transfer framework incorporating Domain-adaptive Neural Network adaptation techniques. Using this resource, key genes related to Cisplatin responses in oral squamous cell carcinoma were identified. Consequently, distinct response patterns were observed in drug-sensitive and drug-resistant cells. Another similar study employed a regularization-based supervised transfer learning technique to predict single-drug response via multi-task learning [47]. Gene expression datasets, including a pharmacogenomic screen, were obtained from GDSC, CCLE and National Cancer Institute’s Dialogue for Reverse Engineering Assessments and Methods drug sensitivity prediction challenge dataset [48]. Furthermore, data from MalaCards [49] were used to identify a subset of genes associated with tissue types of the cell lines [47]. For cytotoxicity prediction, only a few features were required, and the proposed method was reported to outperform previous approaches, including Kernelized Bayesian multi-task learning, Scalable-Time Ridge Estimator by Averaging of Models, and multi-task feature learning. Alternatively, rather than employing ML approaches, a study used traditional statistical methods, specifically survival and objective response rate analyses, to assess monotherapy efficacy in 86 cancer patients [50]. Additionally, the underlying biological mechanisms of neoantigen-specific T-cell expansion in response to therapy were investigated by conducting functional immunological analyses. The study reported observational and mechanistic insights into treatment response. Notably, this study marks the first FDA-approved case in which a cancer drug was prescribed based on biomarkers rather than cancer type, building on earlier work that demonstrated successful translation of drugs into practice.

Recent studies have leveraged statistical modeling and ML to enhance drug response prediction and patient stratification. Zhou et al. (2023) introduced a glioma stemness-associated score (GSScore) by integrating bulk and single-cell RNA-seq data with ML feature selection and multivariate Cox regression [51]. This enabled chemotherapeutic response prediction and subtype classification in lower-grade glioma patients. Fang et al. (2024) developed a hypoxia-related gene signature for cervical cancer using Cox and LASSO regression, which predicted prognosis and cisplatin sensitivity while identifying fostamatinib as a candidate therapy for resistant cases [52]. Similarly, Wang et al. (2023) proposed a pathway mutation signature model that mapped gene mutations to pathways to predict survival and immunotherapy response in melanoma, outperforming tumor mutational burden [53]. Complementing these supervised approaches, Hu et al. (2024) employed unsupervised clustering and weighted gene co-expression network analysis to uncover PANoptosis-related molecular patterns in psoriasis, integrating network pharmacology for drug repurposing [54]. Collectively, these methods demonstrate the versatility of statistical and ML frameworks in capturing complex biological signals for precision oncology.

DL-based strategies have advanced drug response prediction by exploiting high-dimensional transcriptomic data and addressing domain adaptation challenges. Zheng et al. (2023) developed a transfer learning framework that harnesses pharmacogenomic signals from bulk transcriptomes in large cell-line datasets to predict drug sensitivities at single-cell resolution, revealing subpopulation-specific responses and drug-combination effects [55]. Building on this concept, Liu, Duan, and Luo (2024) introduced scAdaDrug, a multi-source domain adaptation model that transfers knowledge from diverse bulk RNA-seq datasets to improve prediction accuracy across single-cell, PDX, and patient cohorts [56]. By employing adaptive importance-aware representation learning, scAdaDrug achieved state-of-the-art performance, surpassing single-source models like CODE-AE, though challenges remain under extreme distributional shifts. These approaches underscore the potential of DL and transfer learning to bridge bulk and single-cell data domains, offering scalable solutions for personalized therapy design.

#### 2.1.2 Single data type with a response matrix

Similar to studies that analyze a single data type with a response vector, cell line experiments have been widely used for drug response prediction but instead focused on a response matrix. These cell line models were utilized in a study to evaluate the efficacy of multiple cancer drugs [57]. A supervised regression approach was employed, integrating biological genotype data and chemical drug structure information using DL techniques. Moreover, data from the Cancer Therapeutics Response Portal (CTRP) v2, Gene Ontology, and the GDSC were utilized to reinforce drug response prediction. In this study, model interpretability was emphasized by identifying key mechanisms and cellular subsystems that contribute to drug response predictions. To support the computational findings, experimental validation was conducted using CRISPR and drug screening. Importantly, the study suggested potential combination therapies, contributing to advancements in precision oncology. In another study, DL approaches were employed, including VAE-based models, to predict drug responses in cancer cell lines [23]. By integrating drug-molecule associations, including gene expression, somatic mutations, copy number variations, and drug-pathway associations, the model captured important transcriptomic features. The study demonstrated strong predictive performance across diverse datasets, improving understanding of biological factors such as the tumor microenvironment and mutational burden that influence drug efficacy. In addition, the model was applied for imputation in drug response analysis. However, challenges arose due to variations in prediction accuracy across drugs, with limited improvement for specific compounds, such as LBW242. Furthermore, VAE-based models underperformed compared to principal component analysis (PCA)-based models in specific model-fitting metrics for some drugs.

Population-based experimental approaches have been utilized to predict drug combination responses. For example, one study analyzed epidemiological data, including diagnostic and procedural details of hospital admissions, computerized prescription records, demographic information, and insurance claims, to assess the effect of proton pump inhibitor use on reinfarction risk [58]. A statistical approach using logistic regression was applied to linked datasets from the Ontario Public Drug Program, the Canadian Institute for Health Information Discharge Abstract Database, the Ontario Health Insurance Plan database, and the Registered Persons Database. Evidence of polypharmacy as a health hazard was reported in the case of Clopidogrel, a drug with a genetically variable therapeutic effect, when used in combination with a proton pump inhibitor. This interaction resulted in a reduced therapeutic effect and an increased vascular risk. These findings underscore the importance of investigating drug combinations.

#### 2.1.3 Multiple data types with a response

Various methods have been employed for drug response prediction using diverse data types. As in previous study types, cell line experiments have been frequently utilized. For example, a network-based approach using data from GDSC was applied to predict drug response [59]. This technique integrated cell line genomic profiles, drug chemical structures, drug-target interactions, and protein-protein interaction data to predict both known and unknown cell line-drug associations. A heterogeneous network-based method was implemented within a supervised learning framework and demonstrated strong protective performance. This approach outperformed traditional regression techniques, as validated through receiver operating characteristic curve analysis, and identified cell line-drug interactions supported by existing literature. Another study utilized gene expression data from GDSC and CCLE, along with clinical trial data and previously published datasets, to develop a supervised classification approach that categorized patients as responders or non-responders based on different clinical criteria, such as response type and tumor regression grade [60]. Deep neural networks (DNNs) were trained to predict drug response, and validation was conducted across various clinical cohorts. Gene set enrichment analysis was performed on weights obtained from the first hidden layer of the optimal neural network. Furthermore, survival analysis was conducted to compare patient groups with the lowest and highest IC50 values, which serve as a measure of drug response [60]. This analysis contributed to addressing the challenge of personalized drug response prediction and highlighted the importance of ML in precision oncology.

Gene expression data were also leveraged in another study [61] from the GDSC and Institute for Molecular Medicine Finland [62] databases, alongside prior knowledge from Functional Linked Networks. The study employed a Bayesian multi-view multi-task sparse linear regression model, integrating multiple input sources to predict drug response in cancer cell lines with improved accuracy. In this supervised analysis, features associated with drug response were determined, providing insights into the mechanism of action of various cancer drugs. Similar to previous analyses, this study explored the relationship between gene expression and drug response in cancer cell lines; however, a dose-varying regression model was utilized [63]. In vitro dose-response and gene expression data from GDSC were used to predict how transcriptomic effects change across distinct drug dosages. A two-stage variable selection method was implemented, beginning with initial variable screening to determine relevant genetic factors followed by penalized regression for further filtering. To validate the model’s predictive accuracy, simulation studies and cross-validation were performed. Furthermore, pathway enrichment analysis was performed to determine biological pathways linked to tumorigenesis and DNA damage response. Multi-Source Drug Response Prediction, a DL framework, was employed in a study for drug response prediction in cancer cell lines [64]. The model incorporated an interaction module to identify pairwise relationships between cell lines and drugs while integrating multiple drug-related associations, such as diseases, targets, and side effects, through similarity network fusion. Enhanced in vitro drug response prediction was achieved by using pairwise interactions and multisource drug features. While the model demonstrated strong predictive capabilities, limitations related to data sparsity, molecular representation, and sample size should be addressed for optimization.

Single-cell RNA-seq, spatial transcriptomics, bulk RNA-seq, and clinical data were integrated by Yang, Lin, and Sun (2023) to validate targeted therapy response predictions in glioma [65]. Using a cohort of 19 glioblastoma patients with matched expression, mutation, and clinical data, they performed survival analyses to assess prognostic value. Compared to single-modality models, this multimodal approach offers stronger biological support and more clinically relevant predictions.

To maximize the predictive value of multi-omics data, different integration strategies have been developed. Early integration concatenates multiple omics datasets into a single feature matrix, which is then used to train predictive models. Intermediate integration projects omics data into shared and modality-specific representations that are then combined for prediction. In contrast, late integration processes each omics layer separately and merges their outputs at the decision stage. These strategies provide complementary ways of capturing biological complexity and advancing drug response prediction (Fig. 3). A recent study employed a novel DL approach that integrated multi-omics data and graph embeddings [66]. A supervised regression framework was applied to data from the CCLE and GDSC databases, incorporating copy number variation, gene expression, mutation, and reverse-phase protein array data, along with prior knowledge from the interactome. The DNN model showed superior performance in predicting drug responses, highlighting the importance of multi-omics integration alongside prior knowledge. Moreover, an attention mechanism was utilized to identify the relative contributions of different omics features. A study also employed the DNN-based framework Precily, alongside other ML methods, such as RF and ElasticNet, to predict drug responses in cancer using multi-omics data from both in vitro and in vivo models [67]. High-throughput datasets from GDSC, CCLE, and the Cancer Therapeutics Response Portal v2 were used for model training, integrating single-cell and bulk RNA-seq data to optimize predictive performance. Precily identified clinically and biologically relevant drug-pathway relationships associated with treatment resistance and sensitivity when tested on prostate cancer cell lines and xenograft models. Moreover, the model successfully predicted responses to previously unseen drugs, including metformin and orlistat, demonstrating its potential for drug repurposing. Further validation using tumor RNA-seq data from TCGA confirmed its applicability in real-world clinical settings, particularly for patients with BRAF-mutant melanoma. In a separate study, VAEs were applied to integrate multi-omics data—including genomics, transcriptomics, proteomics, metabolomics, lipidomics, gut microbiome profiles, and clinical phenotypes—from the DIRECT diabetes cohort [68]. Using in silico perturbations in the learned latent space, the MOVE framework identified drug–omics associations for 20 commonly prescribed diabetes medications. This approach revealed mechanistic insights, such as distinct multi-omic signatures differentiating simvastatin from atorvastatin and novel links between metformin and gut microbiota composition. The study highlights how drug effects are distributed across multiple omics layers, enabling more sensitive and comprehensive detection of drug-associated molecular changes compared to conventional statistical methods.

Few-shot ML was utilized to predict drug responses in cancer models, including cell lines, PDXs, and patient-derived tumor cell cultures in mice [69]. This study employed a supervised transfer learning approach, where large-scale gene expression and somatic mutation datasets from GDSC, CCLE, and the Cancer Dependency Map were initially used to train neural networks and later adapted to new scenarios using limited additional samples. Similarly, zero-shot ML was employed to predict drug responses. This study utilized a Multi-branch Multi-Source Domain Adaptation Test Enhancement Plug-in (MSDA) to predict drug responses for novel compounds using cell line data from GDSCv2 and CellMiner, demonstrating efficient predictions and general performance improvements in preclinical drug screening [70]. Another recent study used gene expression and mutation profile data from the CCLE and GDSC repositories [3]. This study examined the performance of ML and DL models in predicting drug responses in cancer cell lines using a supervised regression framework for 24 individual drugs. ML models, specifically ridge regression, performed comparably to DL models, revealing their potential for explainable AI applications in identifying key genomic predictors of drug response. However, a limitation of this study was that drug response prediction models were trained exclusively on CCLE data, which may affect generalizability of the findings. Another study used a DL model, CDRscan, to predict anticancer drug response using genomic and drug screening data from the COSMIC Cell Line Project and GDSC [24]. To perform the analysis, a supervised ML framework involving regression techniques was employed. Convolutional layers and a virtual docking mechanism were utilized to improve predictive accuracy, achieving an AUROC > 0.98 and R^2^ > 0.84. This highlights CDRscan as a valuable tool for drug repurposing and personalized medicine, facilitating tailored cancer therapy informed by genomic information. However, further in vivo validation is required to confirm its clinical translatability.

### 2.2 Unstructured data

In this section, we consider unstructured data, which are characterized by the absence of a predefined schema or consistent data model, making them difficult to represent in traditional row-and-column formats without loss of information. Unlike structured data, these data do not follow fixed rules or patterns and are often stored in heterogeneous formats, such as free text, emails, reports, images, videos, audio files, computer-aided design models, or scans. Although certain metadata, including pixel values or file attributes, can be captured in structured form, the underlying semantic content usually remains inaccessible without additional processing. Other examples of unstructured data include sensor data, clinical notes, and genomic sequencing information. Table 2 outlines studies employing unstructured data types and their respective designs, with further details available in Supplementary Table 1.

#### 2.2.1 Single data type with a response vector

Image-based experiments were commonly used for drug response prediction, followed by digital health experiments. A previous study utilized an unsupervised ML approach, incorporating a SVM and a dosage-clustering algorithm, to assess drug effects and classify cell phenotypes using high-dimensional image-based data [71]. Using multivariate statistical analysis, the study determined key morphological features that distinguish cellular responses to monotherapy. To further refine the dataset, feature selection techniques were employed to identify the most informative variables, enhancing interpretability and efficiency in drug screening.

A study presented an advanced DL framework that combines a multiple instance learning method, named ‘Noisy-AND’, with CNNs to analyze high-content screening (HCS)-generated microscopy images [72]. An interpretable dataset based on populations of digits from the MNIST handwritten digit dataset [73] was utilized to highlight the effectiveness of this approach in classifying and segmenting cellular populations under various drug treatments. Additionally, ‘Noisy-AND’ pooling was employed to reduce the need for extensive manual labeling, enabling the model to classify images only using image-level annotations. This method outperformed existing approaches on mammalian and yeast datasets, demonstrating its potential for high-throughput cell biology and drug screening applications. Another study extracted feature representations from microscopy images without requiring labeled data by employing a CNN within a self-supervised learning framework [74]. The technique, known as paired cell inpainting, trained the network to predict fluorescence patterns in one cell based on another cell from the same image by reconstructing the appearance of the protein channel in the target cell. As a result, advanced biological analyses, such as proteome-wide clustering and cell variability quantification, were enabled by the model’s ability to reconstruct the appearance of the protein channel in the target cell. Another study also applied CNN techniques, but used a multi-scale approach for phenotypic classification of cellular images [75], utilizing datasets from the Broad Bioimage Benchmark Collection (BBBC013-BBBC021) [76], the Human Protein Atlas [77], [78], and the WND-CHARM collection (HeLa and CHO datasets) [79]. Raw pixel intensity values were directly processed using this supervised DL method, eliminating the need for manual preprocessing, complex multi-step pipelines, or prior knowledge. Additionally, optimization of the network weights was performed using labeled datasets, enabling superior accuracy and simplicity compared to existing methods, thus facilitating both phenotype identification and drug potency analysis. However, the model showed comparatively lower performance on the Human Protein Atlas dataset, and the study’s comparisons were restricted by the available experimental methodologies and scenarios. A study employed a deep metric learning method to analyze image-based screening data from the BBBC021 dataset, focusing on the MCF-7 wild-type P53 breast cancer cell line treated with multiple drugs [80]. Both classification and regression were performed using this supervised learning method, utilizing a pre-trained network and transformations sensitive to biological differences. As a result, the method achieved high sensitivity and robustness without requiring labeled training data, allowing for phenotype classification and continuous similarity measurement. However, the analysis was limited to a relatively small screen of a single cancer cell line, where nuisance factors may have introduced bias. Additionally, a comparison of the study’s Normalized Cytological Similarity Benchmark metric with previous work was not possible due to differences in neural network architectures. Furthermore, the absence of ground-truth data for the correct dose-response curves of each drug presented challenges in validating its predictive performance.

#### 2.2.2 Single data type with a response matrix

Similar to the single data type with a response vector scenario, image-based experiments have frequently been used for drug response prediction in the single data type with a response matrix case [81]. A combination of unsupervised learning methods, including Gaussian mixture modeling [82] and factor analysis [83], has been used for image-based profiling of cellular morphological responses to small-molecule treatments. However, a potential limitation of this method is the exclusion of important, nonredundant image-based features, which has been observed in lower-dimensional screening contexts. On the other hand, a separate study leveraged CNNs in HCS to classify phenotypes, taking advantage of their ability to automatically learn and extract important features from single-cell imaging data [84]. In this study, CNNs significantly reduced the misclassification rate and outperformed traditional ML techniques. Another study also employed CNNs, in this case using them for transfer learning on the BBBC021 image dataset to facilitate morphological profiling of microscopy images [85].

Several studies have also applied statistical analyses to predict drug or intervention responses, particularly in clinical trial-based experiments. For example, a study examined the effects of sacubitril/valsartan treatment on sleep-related endpoints in 140 patients with heart failure with reduced ejection fraction and New York Heart Association class II or III symptoms [86]. Similarly, another study used data from the same clinical trial [86] to assess daily physical activity and sleep patterns in heart failure patients using actigraphy, a wearable device that measures 24-hour activity [87]. However, a key limitation of this study was its short duration, which may not fully capture the long-term impact of therapy on patients’ sleep and activity levels. In a separate study, researchers assessed the implementation and outcomes of a web-based clinical trial investigating the effectiveness of tolterodine ER 4 mg in patients with overreactive bladder [88]. However, the small sample size posed a limitation, potentially restricting the generalizability of the findings. A separate clinical trial evaluated the effectiveness of MEDication reminder APPs in improving medication adherence among patients with coronary heart disease [89]. Additionally, the impact of continuous glucose monitoring compared to standard care was evaluated in a randomized clinical trial including patients with type 2 diabetes requiring multiple daily insulin injections [90]. Statistical analyses, including mixed-effects linear models and logistic regression models, were conducted to assess glycemic control. In a pilot study, researchers investigated the effectiveness and feasibility of the eCO (eCediranib/Olaparib) mobile medical application and provider web portal in managing treatment-related hypertension and diarrhea in patients enrolled in a clinical trial of Olaparib and Cediranib [91].

While the aforementioned studies focused on clinical trial-based experiments, another study investigated phenotype changes associated with disease using an automated image-based approach for drug discovery [92]. Utilizing the Cytometrix™ system, different drug compounds were analyzed through phenotypic profiling, allowing for precise classification based on cellular responses. However, the study also acknowledged challenges, particularly the inability to detect activity in drugs that do not induce significant growth inhibition.

Unsupervised learning techniques have also been applied to unstructured data in studies predicting intervention effects. One such study presented a comprehensive analysis of the genetic and transcriptional heterogeneity within human cancer cell lines, using the MCF7 breast cancer cell line as a case study [93]. Unsupervised hierarchical clustering of 27 MCF7 strains, based on 1784 morphological features, was performed alongside genomic analyses of 106 human cell lines grown in two laboratories to show extensive clonal diversity. Another study employed an imaging-based approach to analyze HCS results using self-organizing maps to group treatments based on morphological similarities [94]. This study presented a novel approach for the analysis and visualization of HCS data, which serves as a powerful tool in lead discovery. However, a key limitation was the difficulty in selecting relevant groups due to the large number of compounds tested in these screens. A proof-of-concept study explored the use of morphological profiling, via a microscopy-based Cell Painting assay, to systematically annotate the functions of human genes and disease-associated alleles [95]. The study employed PCA to reduce dimensionality and hierarchical clustering to group gene overexpression constructs into 25 clusters based on the similarity of their morphological profiles. To enhance the interpretation of these profiles, the researchers identified 20 subpopulations of cells using k-means clustering on the single-cell data. Novel functional associations between critical pathways involved in tumor initiation and progression were determined by analyzing morphological changes caused by gene perturbations. Nonetheless, a key limitation of using RNA interference for morphological profiling is that an excessive number of measurements may generate profiles influenced by off-target effects, particularly seed effects, which can obscure true functional relationships.

#### 2.2.3 Multiple data types with a response

A recent study that employed various data types for drug response prediction proposed a DL framework aimed at improving personalized treatment strategies through multi-omics data integration [96]. The model used CNNs to convert the structured data into images, with distinct colour channels preserving complex inter-omics relationships. Furthermore, the model was trained on GDSC cell line data, with validation on data from TCGA and drug response data from the PDX Encyclopedia. Strong classification performance was demonstrated, with CNNs effectively capturing non-linear patterns that traditional ML methods might miss. The framework also facilitated the detection of genomic factors contributing to drug responses, aiding drug target discovery. However, limitations included a small sample size, which could reduce model reliability and increase the risk of overfitting, as well as concerns regarding the generalizability of in vitro results to real patient data.

The RATE-AF randomized trial investigated the use of consumer wearable devices to evaluate heart rate control in older, multimorbid patients with permanent atrial fibrillation and heart failure [97]. Participants were randomized to treatment with either digoxin or beta-blockers, and heart rate and physical activity data were collected using wrist-worn wearables linked to smartphones over 20 weeks. The study employed self-supervised CNN learning to analyze the large volumes of data generated by the wearables. This method accounted for missing data and predicted New York Heart Association functional class five months after baseline assessment, showing similar accuracy to standard clinical measures such as electrocardiographic heart rate and the 6-minute walk test. The results indicated that digoxin and beta-blockers had equivalent effects on heart rate, suggesting that dynamic monitoring using wearable technology could be an alternative to in-person assessment.

### 2.3 Structured and unstructured data

In this section, we discuss both structured and unstructured data types, which together represent the diverse information used in biomedical research and drug response modeling. Structured data provide standardized, easily searchable formats suited for statistical and computational analyses, while unstructured data capture complex, high-dimensional information such as text, images, and genomic sequences that often require advanced processing for extraction and interpretation. Table 2 presents studies incorporating both structured and unstructured data types along with their study designs, with additional details available in Supplementary Table 1.

#### 2.3.1 Multiple data types with a response

This study presented TGSA, a novel drug response prediction framework, which employed supervised regression techniques to enhance response prediction in cancer cell lines by incorporating gene expression, somatic mutation, and copy number variation data [26]. The model integrated domain knowledge, gene interactions, and cell line/drug similarity information to improve prediction accuracy, utilizing cell line graphs based on STRING protein-protein associations [98] and molecular graphs of drugs derived from atomic structures. The datasets were collected from well-established databases, such as GDSCv2, CCLE, and COSMIC. However, the model has limitations. Constructing cell line graphs based solely on STRING interaction scores overlooks important layers of biological context, including co-expression patterns, regulatory interactions, and cell-type specific interactions. Incorporating more advanced graph representations, such as gene co-expression networks or weighted dynamic edges, could improve the model’s ability to capture biologically relevant relationships.

Beyond these approaches, multimodal based Large language Models (LLMs) further extend this paradigm by enabling knowledge-driven modeling across structured and unstructured domains. Multimodal frameworks can capture complementary information that single-modality models miss, and LLMs can integrate biomedical literature, clinical notes, and molecular data to support context-aware predictions and hypothesis generation [99]. Notably, multimodal LLM-agent systems have demonstrated feasibility in oncology by fusing radiomics, pathology, and guideline text to deliver personalized treatment recommendations [100]. While these applications remain nascent, they highlight the potential for bridging molecular and clinical contexts.

## 3 Discussion

Statistical, ML, and DL methods have been employed in drug response prediction, utilizing both structured data, such as omics data and drug descriptors information, and unstructured data including medical imaging and wearables data. Furthermore, various ML approaches have been applied to predict drug response with varying levels of granularity. Some methods predict drug response separately for each drug, while others incorporate response profiles from multiple drugs to improve the prediction accuracy for a given drug. Methods including simple regression techniques have been utilized to identify drug-specific biomarkers [14]; however, such methods do not capture more complex relationships within the data. Ongoing advancements in the interpretability of artificial neural networks [101], [102] are expected to drive further innovations in drug response prediction methodologies [60], [69]. Another promising approach involves autoencoders, which can effectively learn from smaller datasets. Additionally, commonly referenced databases for drug response prediction tasks include GDSC [11], [12], CCLE [14], as well as SMILES and Morgan fingerprints for molecular representations. While performance differences exist across datasets derived from GDSC and CCLE, research suggests that the relative performance trends of predictive models, particularly DL models, remain consistent across these sources [103], [104].

### 3.1 Pathway from in vitro models to clinical endpoints

Phenotype mapping refers to the systematic alignment of drug response measurements and disease-related traits with their underlying molecular determinants across heterogeneous experimental and clinical datasets. In practice, this spans several interconnected strategies. Cross-dataset harmonization ensures consistent interpretation of phenotypes across studies or platforms, as seen in efforts to align cancer subtypes between resources like TCGA and CCLE. Genotype-to-phenotype association links genetic variants or expression patterns to outcomes such as drug sensitivity or disease risk, providing mechanistic insight into predictive signals. Ontology-based mappings, including the use of controlled vocabularies such as the Human Phenotype Ontology, standardize phenotype definitions and facilitate interoperability between preclinical assays and clinical endpoints. More recent computational approaches project phenotypic labels into latent spaces or graph structures, supporting improved integration, knowledge transfer, and predictive modeling. Together, these strategies enable integration of preclinical and clinical datasets, improve reproducibility, and strengthen the translational relevance of drug response prediction frameworks.

Ensuring that modeling efforts translate into clinically meaningful predictions requires rigorous validation strategies designed to minimize overfitting and prevent artificial inflation of performance metrics. Although k-fold cross-validation reduces sensitivity to dataset-specific variability, standard random splits remain inadequate for drug response prediction because they allow information leakage between training and test sets. Critically, they fail to address drug-identity bias—a phenomenon where models learn to recognize specific compounds rather than underlying biological relationships between molecular features and phenotypic responses. This bias can substantially inflate apparent predictive accuracy in benchmark datasets such as GDSC and CCLE, particularly when training and test sets share identical or chemically similar drugs. Therefore, studies should adopt task-specific stratified validation protocols, such as leave-drug-out, leave-cell-line-out, and combination-wise cross-validation, to obtain more realistic estimates of model generalizability. Alongside these measures, hyperparameter tuning should be performed using nested cross-validation, where the inner loop optimizes hyperparameters and the outer loop estimates final model performance. This approach further mitigates overfitting and yields more robust, reliable estimates of model generalizability.

Beyond internal validation and hyperparameter optimization, cross-dataset evaluation offers a rigorous test of model robustness by assessing transferability across pharmacogenomic resources. However, this approach introduces challenges including domain shift and batch effects, which arise from systematic differences in experimental protocols, assay conditions, and response scales. To minimize these discrepancies and enhance reproducibility, studies should integrate harmonization techniques—such as batch correction methods (e.g., Combat, mutual nearest neighbors)—alongside transfer learning strategies like domain adaptation, where the predictive task remains constant, but underlying data distributions differ. Looking ahead, standardized benchmarks and unified evaluation pipelines will be critical to ensure that cross-dataset validation reflects true generalizability rather than dataset-specific artifacts. Reinforcing these efforts, external test protocols using independent clinic-derived datasets are essential for assessing real-world applicability. Such protocols confirm that models trained on preclinical or benchmark data can perform reliably in heterogeneous patient populations and across diverse clinical contexts. Ideally, external datasets should capture variability in demographics, therapeutic strategies, and disease subtypes, providing a robust foundation for translating computational predictions into actionable clinical insights.

Building on the need for standardized benchmarks and evaluation pipelines, careful selection of evaluation metrics is essential. Measures such as accuracy, root mean square error, and AUROC capture different dimensions of model performance and may introduce biases depending on dataset characteristics and task formulation (e.g., binary classification versus dose-response modeling) [105]. Inappropriate metric selection can create a misleading impression of strong performance while masking poor generalizability, highlighting the need for transparent and rigorous evaluation protocols. Handling class imbalance is another critical consideration, particularly when binary sensitivity thresholds such as IC50 cutoffs produce skewed class distributions. To address this, reporting the area under the precision-recall curve alongside AUROC is recommended, as area under the precision-recall curve provides a more informative measure under imbalance. Additionally, preprocessing techniques such as undersampling, oversampling, or synthetic data generation (SMOTE) can be employed to balance classes, though these methods should be applied carefully to avoid distorting underlying biological variability [106], [107].

### 3.2 Trends, limitations, and trade-offs

A review of the literature reveals recurring methodological trends in drug response prediction studies, with computational approaches adopted to different data types. Supervised learning techniques, particularly DL models, are widely used for analyzing large-scale genomic and transcriptomic datasets, where they excel at capturing non-linear relationships in high-dimensional data. However, these models typically demand large sample sizes and careful regularization to mitigate overfitting and ensure generalization. In contrast, ensemble ML methods, such as RF and gradient boosting, are robust for low- to moderate-dimensional datasets and can effectively integrate multi-omics and clinical data even when sample sizes are limited. Yet, these methods may struggle to capture the complex non-linear dependencies present in large-scale molecular profiles. Unsupervised methods, including clustering and dimensionality reduction techniques, are well suited for patient stratification and the identification of novel drug response subgroups. Their strength lies in revealing latent structure without requiring labeled outcomes, but they are limited in their ability to produce predictive models and are highly sensitive to preprocessing choices and parameter tuning. Approaches that incorporate prior knowledge of molecular structural properties, including molecular docking and physiochemical property-based predictions, offer valuable mechanistic insights into drug–target interactions and potential toxicity, yet these approaches may lack scalability when applied to large chemical libraries or complex multi-omics datasets.

Recent advances highlight the task-specific strengths of emerging architectures. For example, GNNs are particularly effective for modeling relational or networked inputs—such as protein–protein interaction networks or molecular graphs—by capturing connectivity patterns that CNNs may overlook [108], [109]. On the other hand, CNNs are well-suited for grid-like or spatially structured inputs, efficiently detecting local patterns in feature matrices derived from omics data. These distinctions emphasize that no single approach is universally superior; rather, performance depends on the alignment between model architecture, data structure, and sample size.

Aside from choices in model selection, ensuring interpretability of predictive models continues to be a major issue. This challenge is particularly evident in DL-based techniques, where the “black-box” nature of the architecture makes it difficult to trace how individual features influence predictions, thereby constraining both biological insight and clinical applicability [110]. Traditional ML methods used in pharmaceutical research face similar challenges [111], and the interpretability of any model is further limited by the quality and completeness of input data [112]. To address these limitations, explainable artificial intelligence methods such as Local Interpretable Model-agnostic Explanations and Shapley Additive Explanations have been increasingly adopted to identify key features driving model predictions [102], [113]. These approaches can help reveal which molecular or cellular features most strongly influence model-predicted drug responses, thereby improving the transparency and clinical interpretability. Nevertheless, there is a trade-off between model complexity and interpretability, as more complex models often achieve higher accuracy but are inherently harder to explain. Ultimately, improving model interpretability is essential for the clinical translation of drug response prediction models into reliable decision support tools that can guide personalized treatment strategies.

With regards to model interpretability, it is important to mention cheminformatics too, which provides an important complementary layer for drug response prediction by enriching the feature space with either molecular descriptors, structural fingerprints, SMILES strings, and/or graph-based representations of compounds. As highlighted by recent reviews such as Partin et al. (2023) [4] many current models still underuse chemical structure information despite its potential to capture drug-centric variation—such as scaffolds, physicochemical properties, and mechanisms of action—that omics-only approaches may miss. Integrating cheminformatics with molecular or clinical data can therefore improve generalization, support drug repurposing, and strengthen mechanistic insight. In addition, chemical features naturally lend themselves to more explainable modelling by linking predictive signals back to specific substructures or drug–target motifs.

Selecting IC50 thresholds to classify drug sensitivity requires careful consideration of drug-dependent variability. While targeted therapies often exhibit clear separations between sensitive and resistant cell lines, broadly acting agents such as cytotoxic or kinase inhibitors typically display continuous response distributions, making binary thresholds arbitrary and potentially misleading. In large-scale pharmacogenomic datasets, drug identity accounts for much of the IC50 variability, and threshold-based classification can amplify this effect, reducing generalizability to novel drugs or unseen contexts [105]. To overcome these limitations, models should increasingly adopt continuous, quantitative response measures—such as AUROC, area above the curve, or the drug sensitivity score—which integrate responses across multiple concentrations and provide more stable, comparable performance estimates [104], [114], [115]. However, the robustness of AI predictions remains dependent on data quality. Ovchinnikova et al. (2024) showed that standard drug sensitivity metrics often obscure subtype-specific treatment differences; applying simple z-scoring improved both interpretability and predictive performance, underscoring the importance of preprocessing in precision oncology workflows [116]. Additional normalization strategies, such as response scaling or drug-wise aggregation, can further reduce drug-driven biases and enhance reproducibility across datasets [116]. Collectively, these refinements—including rigorous data splitting, domain adaptation, class balancing, and the use of continuous, integrative metrics—represent actionable best practices for developing drug response prediction models that are both robust and generalizable.

While large-scale cell line datasets obtained from resources such as GDSC and CCLE have been instrumental in advancing drug response prediction, they capture only part of the biological complexity underlying treatment outcomes. Two-dimensional culture models exert selective pressure that diminish tumor heterogeneity over time, while genetically engineered mouse models are limited in recapitulating patient-specific disease progression and histological complexity. Patient-derived models, including organoids and PDXs have emerged as more physiologically relevant alternatives, as they preserve the genetic, molecular, and histological features of the original tumors and more faithfully reproduce intratumor heterogeneity and patient-specific treatment responses. Advances in single-cell sequencing further advance this capability by resolving clonal structure and tumor evaluation at high resolution, uncovering subclonal mutations and transcriptional diversity often masked in bulk profiling [117]. In parallel, emerging ML approaches, such as transfer learning and few- or zero-shot learning, offer opportunities to improve model generalizability, particularly when predicting responses for rare drugs, uncommon tumor subtypes, or patient-derived samples with limited labeled data. The integration of patient-derived models, single-cell data, and advanced learning strategies holds promise for enhancing the clinical relevance of drug response prediction and addressing the heterogeneity that drives therapeutic outcomes and patients.

With the growing availability of multi-omics datasets, integration across diverse molecular layers has strong potential for improving drug response prediction; however, there are associated trade-offs. A key limitation is the inconsistent availability and quality of omics data across samples, such as genomics data, which creates difficulties for models that depend on complete profiles. Moreover, integrating heterogeneous datasets increases computational complexity and can hinder interpretability, thereby limiting mechanistic insights. To address these challenges, careful handling of incomplete data through imputation, sensitivity analyses, or alternative modeling approaches are needed to ensure that findings from multi-omics studies remain reliable [118], [119].

Bias in datasets used for drug response prediction also remains a significant challenge, primarily due to the underrepresentation of diverse populations. Many large-scale health datasets, which underpin ML, DL and statistical models, often lack sufficient genetic, ethnic, and socio-environmental diversity necessary for equitable healthcare outcomes, thereby limiting their applicability across different patient groups. This lack of representation, known as “Health Data Poverty”, highlights how marginalized populations are excluded from data-driven advancements, including AI-driven drug response prediction models [120]. Consequently, biased algorithms may yield less accurate predictions for underrepresented groups, further exacerbating existing healthcare disparities. Ensuring that datasets are diverse and representative of the populations they aim to serve is essential for developing equitable and reliable drug response prediction models [121].

Multimorbidity, where patients present with multiple coexisting diseases, introduces further variability in disease progression and treatment outcomes, thereby complicating drug response prediction. Polypharmacy, or the concurrent use of multiple medications, adds another layer of complexity by increasing the risk of drug-drug interactions that may alter therapeutic efficacy or lead to adverse effects. Key confounding factors, such as age, sex, and comorbidities, influence drug metabolism through pharmacokinetic and pharmacodynamic variations. In ageing populations, for example, physiological changes affecting drug absorption, distribution, metabolism, and excretion increase the risk of unpredictable drug responses and interactions, as highlighted in previous studies [122].

### 3.3 Directions for future research

Drug response prediction relies on the use of diverse data types to comprehensively capture the complex factors influencing treatment outcomes. These data sources typically include genomic, transcriptomic, and proteomic profiles, which provide insights into the distinct, or sometimes overlapping, layers of genetic variations, gene expression patterns, and protein interactions that regulate drug efficacy [123]. Most studies have utilized genomic or transcriptomic profiles of cancer cell lines, in conjunction with drug profiles, for drug response prediction using ML. Moreover, comparative studies indicate that among individual data types, gene expression demonstrates the strongest predictive performance, with only slight improvements when additional data types are incorporated [48], [114], [124], [125]. For example, Iorio et al. (2016) found gene expression to be the most effective standalone predictor across multiple cancer types [126]. Further comparative studies for other heterogeneous diseases, such as hypertension, are required to determine the data types that offer the best predictive performance. The trend in genomic data use persists despite the expectation that proteomics and phosphoproteomics data would be more suitable, given that drugs primarily target proteins rather than DNA or RNA [127]. A major factor contributing to this discrepancy is the limited availability of large-scale datasets that include proteomics and phosphoproteomics data with an adequate number of cell lines to support robust drug response prediction. However, the recent availability of a proteomics and phosphoproteomics dataset with a sufficient number of cell lines [128], [129] presents an opportunity to further investigate the potential of these modalities, which remains largely unexplored.

The integration of multi-omics data is essential for improving the robustness of biomarker discovery. In this context, metabolomics and microbiome data provide complementary layers of information that enhance our understanding of inter-individual variability in drug outcomes. Baseline metabolic profiles have demonstrated utility in predicting drug efficacy and safety by capturing individual differences shaped by genetic, microbial, and environmental factors [130], [131]. Moreover, tracking metabolite changes post-treatment enables the real time assessment of therapeutic efficacy [132]. The integration of metabolomics with additional omics layers, such as genomics and transcriptomics, facilitates the elucidation of biological mechanisms underpinning heterogeneous drug responses. Likewise, incorporating microbiome data into predictive models allows for the consideration of host-microbiota interactions, which play a key role in modulating drug metabolism and pharmacodynamics [133]. Despite the availability of extensive datasets, random and systematic variability across datasets remains a significant challenge in drug response prediction. This further complicates the clinical utility of ML applications. Yet, ML will undoubtably play a major role in shaping the future of drug prioritization in clinical practice [134]. The growing availability of large-scale, curated datasets is expected to further advance the development and application of drug response prediction models. As multi-omics data become increasingly accessible, future studies are likely to incorporate these diverse molecular layers to capture the complex biological interactions underlying drug sensitivity and resistance. Such integrative approaches will be crucial for extending drug response prediction beyond traditional cell line studies and toward more physiologically relevant preclinical and clinical models.

To enhance drug response prediction studies, researchers can harness the dynamic nature of disease progression by utilizing longitudinal data, which capture repeated measurements over time. Moreover, advanced statistical models designed for longitudinal data analysis can be employed to identify key time-dependent biomarkers and develop models that account for the evolving relationship between treatment and response. These approaches facilitate a more nuanced understanding of individual patient trajectories, potentially yielding more accurate drug response predictions as demonstrated in previous studies, compared to analyses based on single-timepoint data.

Future research should prioritize developing robust multimodal frameworks that integrate structured molecular profiles with unstructured data sources, as well as leveraging large language models (LLMs) for knowledge-driven modeling. These approaches can enable context-aware, interpretable predictions and support longitudinal modeling of drug response, representing a major step toward personalized and clinically actionable precision medicine [99], [100], [135].

## 4 Conclusions

To optimize treatment outcome prediction, ML, DL, and statistical modeling, in conjunction with multi-omics, clinical and molecular data, have made significant contributions. Despite these advancements, challenges remain in enhancing model interpretability, generalizability, and clinical translation. Addressing these limitations through careful trade-off analysis and integrating task-appropriate models with phenotype mapping, standardized evaluation, and cross-dataset validation will improve reproducibility and clinical applicability. Interdisciplinary collaboration among computational scientists, clinicians, and pharmacologists is crucial to facilitate knowledge exchange and integrate diverse expertise. This collaborative approach will drive the development of more reliable, interpretable, and actionable models that support precision medicine. Future research should prioritize robust data integration, standardized methodologies, evidence-based strategies to ensure clinical applicability and trustworthiness. Finally, emerging multimodal LLM-based frameworks offer a promising avenue for integrating structured omics with unstructured clinical data, enabling context-aware predictions.

## CRediT authorship contribution statement

**Animesh Acharjee:** Writing – review & editing, Writing – original draft, Supervision, Project administration, Methodology, Investigation, Funding acquisition, Conceptualization. **Georgios Gkoutos:** Writing – review & editing, Writing – original draft, Supervision, Resources. **Durga Parkhi:** Writing – review & editing, Writing – original draft, Investigation, Formal analysis. **Xin Guan:** Writing – review & editing, Writing – original draft, Investigation, Formal analysis. **Yuanwei Xu:** Writing – review & editing, Writing – original draft, Investigation, Formal analysis, Data curation. **Laura Bravo-Merodio:** Writing – review & editing, Writing – original draft, Methodology, Investigation, Data curation. **Daniella Okyere:** Writing – review & editing, Writing – original draft, Visualization, Software, Methodology, Investigation, Formal analysis, Data curation.

## Funding

Funded by Hypermarker EU and MRC Heath Data Research UK (HDRUK/CFC/01) initiatives funded by UK Research and Innovation, Department of Health and Social Care (England) and the devolved administrations, and leading medical research charities. The views expressed in this publication are those of the authors and not necessarily those of the NHS, the National Institute for Health Research, the Medical Research Council, or the Department of Health.

## Declaration of Competing Interest

The authors declared that they do not have any competing interests.

## Glossary

Area under the receiver operating characteristic curve: global performance metric that represents the likelihood that a randomly selected positive instance will be ranked higher than a randomly selected negative instance.
Artificial intelligence: branch of computer science that involves developing algorithms that replicate perceptual, cognitive, and conversational abilities typically associated with human intelligence.
Deep learning: specialized branch of machine learning that employs artificial neural networks modeled after the human brain to simulate complex patterns of computation and cognition.
Deep neural networks: a type of artificial neural network that contain multiple hidden layers between input and output layers.
High-content screening: microscopy-based technique that captures and processes high-dimensional cellular images to quantify morphological and phenotypic traits.
Machine learning: subfield of artificial intelligence that enables algorithms to automatically learn meaningful relationships and patterns from data.

## Appendix A Supplementary material

Supplementary Table 1 Summary of drug response prediction studies, outlining the data types used, analytical approaches (statistical, machine learning, or deep learning), and computational tools developed

## Footnotes

Appendix A Supplementary data associated with this article can be found in the online version at doi:10.1016/j.csbj.2025.11.067.
